# A Functional Neuroimaging Study of Sound Localization: Visual Cortex Activity Predicts Performance in Early-Blind Individuals

**DOI:** 10.1371/journal.pbio.0030027

**Published:** 2005-01-25

**Authors:** Frédéric Gougoux, Robert J Zatorre, Maryse Lassonde, Patrice Voss, Franco Lepore

**Affiliations:** **1**Centre de Recherche en Neuropsychologie et Cognition, Département de PsychologieUniversité de Montréal, Montréal, QuébecCanada; **2**Neuropsychology/Cognitive Neuroscience Unit, Montreal Neurological InstituteMcGill University, Montreal, QuébecCanada; **3**Institut Universitaire de Gériatrie de MontréalMontréal, QuébecCanada; Washington University in St. LouisUnited States of America

## Abstract

Blind individuals often demonstrate enhanced nonvisual perceptual abilities. However, the neural substrate that underlies this improved performance remains to be fully understood. An earlier behavioral study demonstrated that some early-blind people localize sounds more accurately than sighted controls using monaural cues. In order to investigate the neural basis of these behavioral differences in humans, we carried out functional imaging studies using positron emission tomography and a speaker array that permitted pseudo-free-field presentations within the scanner. During binaural sound localization, a sighted control group showed decreased cerebral blood flow in the occipital lobe, which was not seen in early-blind individuals. During monaural sound localization (one ear plugged), the subgroup of early-blind subjects who were behaviorally superior at sound localization displayed two activation foci in the occipital cortex. This effect was not seen in blind persons who did not have superior monaural sound localization abilities, nor in sighted individuals. The degree of activation of one of these foci was strongly correlated with sound localization accuracy across the entire group of blind subjects. The results show that those blind persons who perform better than sighted persons recruit occipital areas to carry out auditory localization under monaural conditions. We therefore conclude that computations carried out in the occipital cortex specifically underlie the enhanced capacity to use monaural cues. Our findings shed light not only on intermodal compensatory mechanisms, but also on individual differences in these mechanisms and on inhibitory patterns that differ between sighted individuals and those deprived of vision early in life.

## Abstract 2

Blind individuals who localize sound better than sighted subjects recruit visual cortical areas in the process

## Introduction

Animals and humans deprived of vision have been shown to have enhanced nonvisual perceptual abilities. Indeed, many blind individuals are extremely efficient in tactile processing, including Braille reading; the talent manifested by some well-known blind musicians, singers, and even piano tuners is often in part attributed to the fact that they were blind since their youth. One may conclude that blind persons should be better in nonvisual tasks since they compensate for their lack of vision by focusing on their remaining modalities. Many studies have in fact shown that some early-blind human subjects outperform sighted persons in nonvisual tasks, such as speech perception [1,2,3], unfamiliar voice recognition [4], verbal memory [5,6,7], and musical abilities [8,9,10]. Of particular relevance to the present study are data suggesting that some blind individuals show better auditory spatial discrimination [11] or localization of sound sources than sighted subjects [2,12,13]; however, other studies have failed to show this advantage [14,15], raising the question of what may underlie individual differences. In general, the nature of any behavioral enhancement, its extent, and its neural bases are still matters of considerable debate.

Animal studies provide some insight as to the neural substrates underlying such enhanced capacities (reviewed in [16]). For instance, in cats that had been visually deprived for several years by eyelid suture shortly after birth, the auditory cortical representation expanded into visual areas [17], and auditory spatial tuning was sharpened in the auditory cortex [18]. Similarly, in neonatally enucleated rats, electrophysiological recordings showed somatosensory responses in the visual cortex [19], and the somatosensory cortex showed an enlargement of receptive fields of the cells in some barrels together with an increase of angular sensitivity for deflection in another barrel [20]. Thus, those experiments indicate a recruitment of the visual cortex for nonvisual tasks, but do not conclusively prove that the enhanced perceptions of the blind rely on the visual cortex.

Several studies using neuroimaging techniques have also established that posterior visual areas in blind individuals may be active during the performance of nonvisual tasks such as Braille reading [21,22], memory retrieval [7], and auditory localization [23,24] as well as other auditory functions [25,26,27,28,29]. It remains to be established whether recruitment of visual cortices necessarily reflects functional reorganization, or whether it indicates a nonspecific or even pathological response. Indeed, despite numerous studies showing activation in visual areas during nonvisual tasks, the functional significance of this phenomenon has been questioned by some investigators who suggest that the occipital cortex might be nonspecifically coactivated [30]. If the visual cortex participates in nonvisual functions in the blind, then its activity level should be related to individual differences in behavior, and in effect predict behavioral outcome.

Localization of sound, a very important function for the blind, is one domain in which it is particularly useful to study the cross-modal interactions that may occur following visual deprivation. This task entails integration of binaural and monaural cues to derive spatial information. In accordance with the idea that nonvisual processing can be enhanced in the blind, a prior study demonstrated that a subset of early-blind subjects was more accurate than sighted controls (SIG) at localizing sound sources, specifically when using monaural cues [12]. These findings provide a clear opportunity to study the nature of visual cortical recruitment, and the extent to which it relates to behavioral improvements.

Thus, in the present study, subjects were first studied in an anechoic chamber using binaural and monaural sound localization tasks. Depending on their performance at the monaural task, they were divided into three groups: (i) early-blind participants who could localize the sounds more accurately than control subjects (early blind with superior performance [EBSP]); (ii) early-blind participants who were unable to localize the sounds any more accurately than controls (early blind with normal performance [EBNP]); and (iii) SIG. The same localization task was next adapted so that it could be carried out within the positron emission tomography (PET) apparatus, using a speaker array which permitted pseudo-free-field presentations [31]. Two control conditions, for monaural sound localization (MSL) and binaural sound localization (BSL), were used to control for the auditory input and motor responses.

The hypothesis tested was that blind persons showing supranormal performance do so because they recruit visual cortical areas to carry out the task. We therefore predicted that they would show activation in visual areas specifically during the MSL task, and not during the binaural or control tasks. The other blind group, which does not have enhanced MSL ability, should not show this activation pattern. We further hypothesized that the degree of visual cortical activity would be predictive of individual differences in the behavioral performance of the monaural task.

## Results

### Anechoic Chamber Experiments

**Figure 1:**
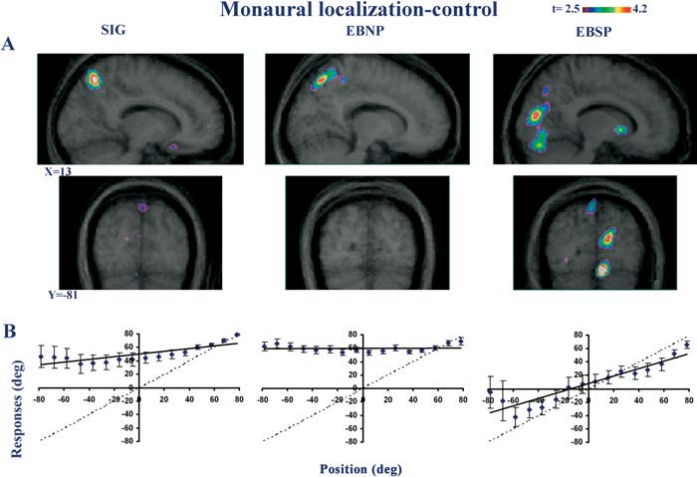
Monaural Sound Localization in PET Experiments Performed in the Three Groups of Subjects (A) CBF increases. Activations of the right striate and extrastriate cortices are observed in EBSP but not in the two other groups for the contrast of MSL minus its control task. Upper image series, sagittal slices; lower image series, coronal slices. X and Y coordinates refer to standardized stereotaxic space. (B) Behavioral data. Behavioral results in MSL task (with SE bars). The dashed lines represent the ideal performance, whereas the solid lines indicate the best linear fit to the observed localization performance. Negative angles on the abscissa correspond to the obstructed ear, while positive angles correspond to the unobstructed ear. Note the better performance of the EBSP group compared to the EBNP and SIG.

In the anechoic chamber experiments, only five of the 12 early-blind subjects could accurately localize the sounds monaurally, whereas most of the sighted subjects could not (Figure 1). In order to differentiate two groups of early-blind participants on the basis of their performance at this monaural task, we computed their mean absolute error score and set the cut-off at a score of 45° (see Materials and Methods). Individuals with scores below this value formed the EBSP group, while those above it constituted the EBNP group. The results of the former group are illustrated on the right side of Figure 1B. As can be seen, in these subjects the regression line was very close to the dashed line representing ideal performance. By contrast, the other subgroup of early blind subjects, EBNP, and the SIG could not localize the sounds correctly, and the regression line is quite distant from that representing ideal performance. To confirm this localization performance, the mean absolute error score was compared for the three groups; a group X position interaction was observed (F_30, 225_ = 6.299, *p* < 0.01), localization being more accurate in the EBSP group, especially on the side of the obstructed ear. The performance of the EBNP did not differ from that of the SIG. These findings confirm the previous study of Lessard et al. [12] with a different group of early-blind participants.

**Figure 2:**
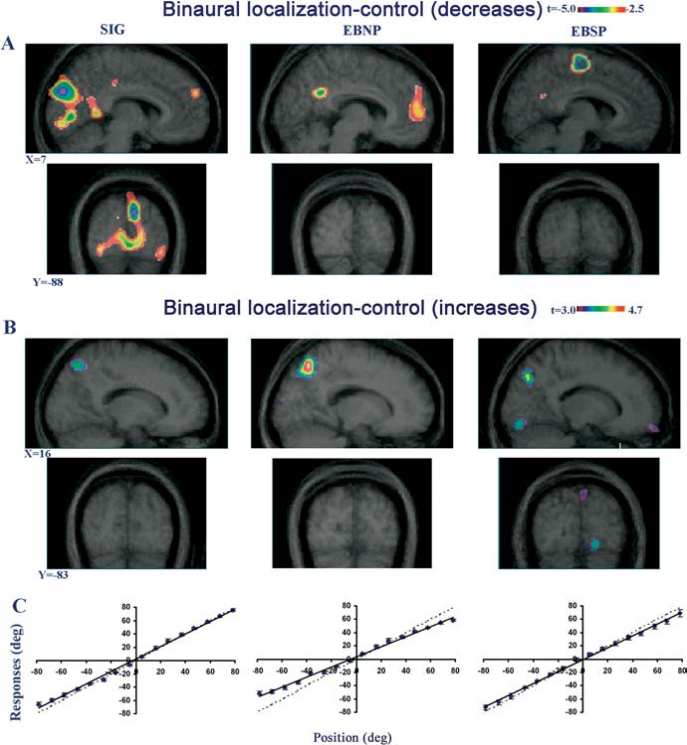
Binaural Sound Localization in PET Experiments Performed in the Three Groups of Subjects (A) CBF decreases. In the sagittal (upper image series) and coronal (lower image series) slices, a decreased CBF is observed in the visual cortex of SIG (striate and extrastriate cortices), for the contrast of BSL minus its control task. X and Y coordinates refer to standardized stereotaxic space. (B) CBF increases. In the sagittal (upper image series) and coronal (lower image series) images, a CBF activation peak is seen in the right ventral extrastriate cortex for the EBSP group, but not for the other two groups, for the contrast of BSL minus its control task. (C) Behavioral data. Behavioral results in the BSL task are presented (with SE bars). The dashed lines represent the ideal performance, and the solid lines indicate the best linear fit to the observed localization performance. All three groups were able to localize sounds accurately.

In contrast, all 19 subjects tested, whether sighted or early blind, could correctly localize sounds binaurally (Figure 2). Despite an overall good performance, as measured by the absolute error score, the EBNP group was somewhat less efficient than the other two groups, especially for more lateral positions (group X position: F_30,225_ = 4.058, *p* < 0.01; see Figure 2C). The latter finding is reminiscent of the undershooting performance also found in visually deprived cats [32].

### PET Scanner Experiments

#### Binaural sound localization

**Figure 3:**
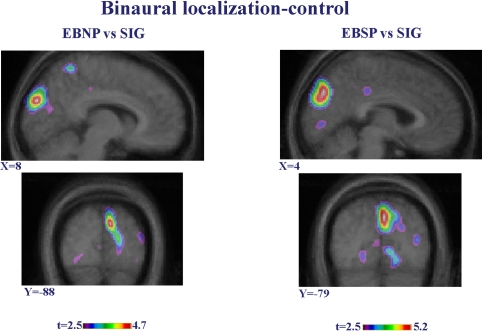
Intergroup Contrasts in Binaural Sound Localization Minus Control Task Sagittal (top) and coronal (bottom) images showing the contrasts between EBNP (left) compared to SIG, and EBSP (right) compared to SIG. These contrasts confirmed the differences in occipital areas between the SIG and the two other groups, which are likely attributable to a decrease in CBF activity in the sighted relative to the control task (see Figure 2). X and Y coordinates refer to standardized stereotaxic space.

During binaural localization of the sound, when compared to the control task (Figure 2A and Table 1), cerebral blood flow (CBF) decreased in the extrastriate and striate cortex of SIG, suggesting inhibition between visual and auditory areas. However, neither group of early-blind subjects showed this deactivation. A further confirmation of these results was obtained by carrying out a direct intergroup comparison of the activation of each of the blind groups to that of the sighted one. These differences are presented in Figure 3. The two blind groups show what appears to be an increase in CBF relative to the SIG, confirming that the CBF response of these groups differ in this region. However, given the deactivation observed within this region in the SIG in the first analysis, as opposed to the lack of CBF difference in the blind, the more likely interpretation is that this effect reflects a decrease in CBF in the sighted.

**Table 1:**
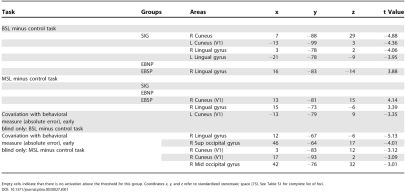
Stereotaxic Coordinates and *t* Values of Activation and Deactivation Foci in Occipital Areas

Empty cells indicate that there is no activation above the threshold for this group. Coordinates *x, y,* and *z* refer to standardized stereotaxic space [75]. See Table S1 for complete list of foci

Another finding from the binaural versus control task contrast was a small region of activation in the visual cortex (ventral extrastriate) of the EBSP group, but not in the other two groups (Figure 2B and Table 1). Finally, all three groups also showed activation in several other cortical regions (see Table S1 for further details); among the most relevant of these is a focus in right inferior parietal cortex (Figure S1).

#### Monaural sound localization.

**Figure 4:**
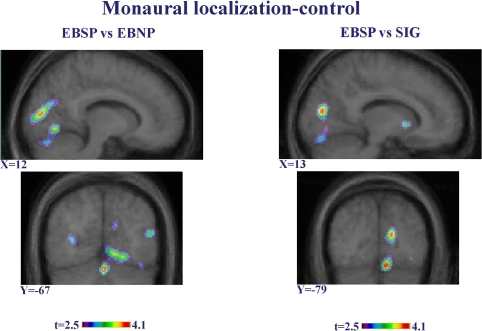
Intergroup Contrasts in Monaural Sound Localization Minus Control Task Sagittal (top) and coronal (bottom) images showing contrasts between the EBSP and EBNP (left), and between the EBSP and SIG (right). These contrasts confirmed the differences in occipital areas between the EBSP group and the two other groups. X and Y coordinates refer to standardized stereotaxic space.

Of greatest relevance to our hypotheses, during monaural stimulation (one ear plugged), as compared to control task, right-hemisphere striate and ventral extrastriate areas showed increased CBF only in the EBSP subset of blind subjects (Figure 1A and Table 1). For the EBNP subset, occipital activation was not significant, as was also the case with the SIG. Once again, direct intergroup contrasts confirmed the differences in activation in occipital areas during MSL between the EBSP group and the two other groups (Figure 4).

While parietal and frontal activations were also seen in the three groups, temporal activations were not found for monaural stimulation, the EBNP group showing even some deactivation in temporal areas. The EBSP group also showed some activation in the right cerebellum (see Table S1 for further details). Significant differences between groups were not observed in temporal and parietal cortices, however.

#### Correlation analysis

**Figure 5:**
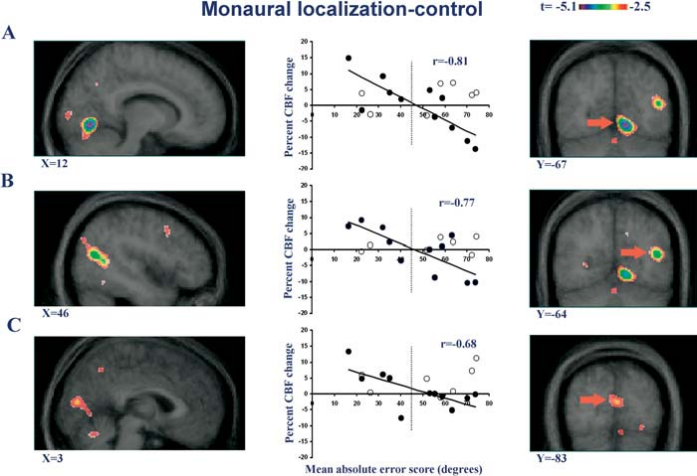
Correlational Analysis for Monaural Sound Localization in Blind Persons These data show the correlational analysis between performance (mean absolute error) in pointing task to monaurally presented sounds and CBF in a group of blind subjects. The two columns of brain images (left image series, sagittal sections; right image series, coronal sections) illustrate the statistical parametric map of the correlation, which is maximal in the ventral extrastriate cortex (A) but also significant in dorsal extrastriate (B) and striate (C) cortices. The red arrows in the coronal slices indicate the focus selected for the respective sagittal slices. The scattergram shows the individual values extracted from each of these regions; closed circles indicate blind subjects; open circles indicate SIG. The dotted vertical line represents the cutoff in performance for the a priori classification of blind subjects into those with low error rates (EBSP) and those who do not show the enhancement (EBNP). X and Y coordinates refer to standardized stereotaxic space.

In order to assess whether occipital activations have a functional role in auditory localization, independent voxel-wise covariation analyses were carried out across the entire group of blind individuals. Irrespective of the group to which they had been assigned, the individual absolute error score was entered as a regressor in the analysis examining covariation with CBF change between overall accuracy at the localization tasks and activation across the entire brain volume, following the procedure outlined by Paus et al. [33]. For MSL, a negative and significant correlation was observed between the absolute error score and CBF in some areas of the visual cortex (especially extrastriate but also striate [Figure 5 and Table 1]). It follows from these results that the degree of activation (percent CBF change) predicted behavioral performance in MSL. The highest correlation observed was in the right ventral extrastriate cortex (lingual gyrus; Brodmann area [BA]18, *r* = –0.81, *p* < 0.01) but two other significant foci were found in right dorsal extrastriate cortex (superior occipital gyrus; BA19, *r* = –0.77, *p* < 0.01), and striate cortex (BA17, *r* = –0.68, *p* < 0.05). Two of these foci are close to the ones identified in the analyses presented for MSL. These findings support the hypothesis that the visual cortex is directly involved in localizing a sound stimulus in the monaural condition.

## Discussion

The imaging results of this study support our hypothesis that blind persons recruit occipital areas in the context of auditory localization and, more importantly, the correlation observed with MSL performance strongly suggests that individual differences in reorganization of the occipital cortex have behavioral consequences. Hence, this relationship does not support the possibility that the recruitment is a nonspecific coactivation or a pathological response. Instead, these results suggest that visual cortex is specifically recruited to process subtle monaural cues more effectively.

Functional activation of the visual cortex by nonvisual stimulation in the blind has already been shown in several previous studies. Activation of primary and secondary visual areas was observed during Braille reading and other tactile discrimination tasks in early-blind persons [21]. This tactile-induced activation in the occipital cortex was also confirmed by a series of subsequent studies [7,22,34]. The hypothesis proposing a functional role for this activation in the visual cortex was supported by the study of Cohen et al. [35] who showed, using transcranial magnetic stimulation, that this occipital area is required for Braille reading in blind subjects. This phenomenon was further illustrated in the case of a proficient Braille reader, blind since birth, who became unable to read Braille, despite normal somatosensory perception, after bilateral occipital damage resulting from an ischemic stroke [36]. Moreover, in a speech processing study in the blind, it was shown that occipital activity (striate and extrastriate) varied as a function of semantic or syntactic content [29]. Activation of area V1 has also been found to correlate with performance in memory tasks [7].

Of greatest relevance for the present findings are the results of Weeks et al. [24] in which CBF was measured during BSL in blind and sighted individuals, and reported activation in posterior parietal areas and also in association areas in the right occipital cortex only in the blind. In addition, interregional covariations observed between the right parietal and occipital (ventral, dorsal, and parieto-occipital) cortices were interpreted as reflecting parts of a functional network for auditory localization [24]. These data and the present findings converge on the conclusion that the visual cortex is recruited during auditory localization in the blind. However, whereas the study of Weeks et al. [24] reports extensive recruitment of visual areas during binaural processing, we observed only a small area of activation in the ventral visual area in the binaural condition. Instead, our data point to the importance of visual regions in successful localization under monaural conditions, which we interpret as reflecting a recruitment of these areas for processing of spectral cues. One possible interpretation of the discrepancy is that the BSL task of Weeks et al. [24] may have involved spectral cues, provided by the head-related transfer functions used in that study to simulate extrapersonal space.

### Functional Significance of the Recruitment of Visual Areas in the Blind: Better Use of Spectral Cues?

It is interesting to consider the function of the right occipital areas, which seem to be important for MSL. Monaural cues (spectral cues and head shadow effect) are involved in the localization of sounds when one ear is obstructed, or in unilaterally deaf subjects [37]. However, spectral cues also contribute to BSL, particularly for vertical and front-back discrimination [38], but also for azimuth localization [39,40]. Moreover, some authors suggest that spectral cues based on head-related transfer function templates are sensitive to experience [41,42]. In this vein, Doucet and coworkers [43] showed that the supranormal performance of early-blind persons in MSL was decreased by occlusion of the pinna or by high-pass and low-pass filtering of the stimuli, again suggesting that use of spectral cues is important for this task. However, because performance was not completely abolished, head shadowing cues [37] might also have operated. Similarly, the study by Röder and collaborators [13] suggests that blind persons might be more sensitive to spectral cues, since their blind participants were better at localizing at lateral positions. Finally, a recent study showed superior binaural spatial discrimination performance in both early- and late-blind subjects compared to sighted subjects, when the stimuli were presented in peripheral field [44]

### Combination of Intramodal and Cross-Modal Plasticity?

Cross-modal plasticity may not necessarily be the only mechanism to explain the present results. Some studies have also shown intramodal plasticity in auditory cortex of the blind. For example, the tonotopic region of area A1 in blind persons seems to be enlarged compared to that of sighted subjects [45], presumably reflecting greater use of auditory cues by the blind. Enhanced recruitment and sharpening of spatial tuning of auditory cortical neurons has also been found in binocularly deprived cats [17,18]. Thus, a combination of intramodal plasticity in auditory cortex and cross-modal plasticity involving visual cortex may have contributed to the superior performance seen in our early-blind subjects. In the present study, a significant difference in activation in auditory cortical areas was not observed among the three groups. However, we cannot exclude the possibility of plasticity at this level, as CBF responses might not be sensitive to effects such as better spatial tuning properties of auditory neurons.

Experience-driven improvements in auditory localization can occur without necessarily invoking cross-modal recruitment. Indeed, some studies have shown that in MSL tasks, practice may lead to increased performance in the case of unilaterally deaf patients [46] or even normal subjects [47]. Excellent performance in MSL was also observed in juvenile ferrets when they were raised with one ear plugged. Even in adult ferrets, changes were seen in the performance during MSL after regular practice [48]. Moreover, adult humans seem to be able to calibrate auditory cues after their pinnae were modified with moulds, showing good performance with their “new ears” after a few weeks [49]. These results would favor the hypothesis that, instead of becoming supranormal in their remaining senses, blind persons may use them more efficiently within normal limits [50]. Nonetheless, our data suggest that this efficiency gain in the blind is achieved at least in part via recruitment of visual cortical areas.

Why do some blind persons and not others acquire superior monaural sound localization skills? It may be that these changes are entirely experience-driven. That is, some blind persons may have had more practice navigating or using auditory cues to explore their environments. On the other hand, the individual differences we observed in the degree of cross-modal plasticity could reflect innate factors that remain to be identified. An additional explanation may be that the blind persons who are not better than normal at sound localization may be superior in other nonvisual tasks, such as Braille reading or other somatosensory discrimination, because the visual cortex was preferentially recruited to carry out these tasks instead of auditory ones. If this is the case, it is possible that cross-modal plasticity is limited to a certain extent, such that recruitment of these areas by one modality inhibits recruitment by another. These speculations will have to be explored systematically in future studies.

### How Does the Visual Cortex of the Blind Process Auditory Information?

What is the nature of the mechanism implied in the processing of auditory stimuli by visual cortical areas? The specific areas of visual cortex recruited may provide a clue. The analyses yielded one peak in the right V1 area. V1 has already been shown to be activated in other studies examining Braille reading [21,22,34], verbal memory [7], verb generation [7,28], and speech processing [29]. These findings therefore suggest that V1 may play a very general role in a variety of nonvisual tasks in the blind. However, a right lingual gyrus peak was the main focus revealed by the analyses in the present study. This cortical region is known to form part of the ventral visual pathway, which is important for identifying visual objects [51]. If this region is important for the processing of visual object features, such as contour or texture [52,53], we may speculate that the same area is possibly used in the blind to process analogous features for auditory stimuli such as spectral contour. These cues to auditory object identity, which are normally processed in anteroventral regions of the auditory cortex [54], might be processed in the occipital ventral stream in the blind when they are relevant for spatial position.

### Contribution of Parietal Cortex to Binaural Sound Localization

The parietal activation observed in all groups during BSL suggests that these areas are important when carrying out the task used in the present experiment (see Figure S1). This finding agrees with a previous study, which reported a right-sided inferior parietal activation that positively correlated with absolute error score in normal sighted subjects with the same procedure as in our study [31]. Parietal activation of both hemispheres, or a right hemisphere advantage, has been shown in several other neuroimaging studies of auditory localization and spatial discrimination experiments with sighted subjects [24,55,56,57,58,59,60]. In the study of Weeks et al. [24], a strong right-hemisphere recruitment of parietal and occipital regions was shown for blind subjects. Our findings therefore agree with these studies and with the well-known right-hemisphere advantage for spatial processing. However, we did not observe preferential activation in this parietal region in the blind as compared to the sighted, nor did CBF correlate with behavioral performance in this region. Based on those findings, we conclude that parietal area activation is related to the sensory-motor integration and spatial coordinate transformation required by the pointing task [31,61] at some stage after sensory processing has occurred. Thus, we propose that in those blind subjects who have specifically learned to use monaural cues, parietal regions receive additional input from the ventral visual cortex, but that no reorganization within the parietal cortex itself has occurred.

### Different Inhibitory Patterns for the Visual Cortex in Blind and Sighted Persons

During BSL, the sighted control group (SIG) showed a deactivation in both extrastriate and striate areas of the occipital lobe, a phenomenon that was not observed in either subset of early-blind individuals. Many previous studies with sighted subjects have shown that following stimulation in one modality, cross-modal inhibition occurs in the unattended modalities [62,63,64,65,66] or even in some areas within the same modality [66,67]. Interestingly, an imaging study with sighted subjects carried out by Zatorre and coworkers [57] reported a visual deactivation in tasks of pitch and location discrimination. Because deactivation is not seen in all studies, the phenomenon may be related to the nature of the task [64,65,68].

Deactivation of primary visual areas has also been seen in sighted subjects during a tactile discrimination task, whereas in blind subjects activation was shown in the same area [21]. Within the context of auditory localization, Weeks and coworkers [24] also reported some occipital deactivation in sighted subjects, while the blind showed activation in the same area. All these results suggest that cross-modal inhibitory processes could be different in blind and sighted subjects, at least under some experimental conditions. Blind subjects might not have to inhibit the normally competing visual cortex when they perform some of the same nonvisual tasks as sighted people do. By contrast, the specific recruitment of the same cortex in order to complete a difficult task might permit them to compensate for their handicap.

### Conclusion

The present study establishes for the first time in certain early-blind persons a clear relationship between monaural sound localization performance and increased CBF in occipital areas. Indeed, some of the blind persons showed occipital activation that appeared to be functional, since this phenomenon was correlated with a supranormal performance in MSL. This finding suggests that visual deprivation from an early age could lead to important cross-modal plasticity and give blind persons an advantage in using spectral cues to carry out a crucial everyday task, sound localization. Moreover, we report that inhibitory patterns differ between early-blind and sighted individuals. Under binaural conditions, the SIG seemed to inhibit part of the occipital areas when localizing sounds, but this was not the case for either group of blind persons. This differential pattern may provide clues as to how different parts of the brain normally interact during unimodal stimulation, and further suggests that these interactions may be modified in the absence of a sensory modality.

It may also be important in future studies to investigate whether blind persons can recruit visual areas in other auditory tasks, for example in a task in which spectral and level cues are relevant but in a nonspatial context. Along the same lines, a spatial discrimination task not requiring the explicit localization of the sounds may also be of interest. One can thus verify that this special competence of some blind persons can be generalized in different auditory contexts other than MSL. Indeed, it would be interesting to know whether this ability is related to more complex tasks such as navigation, obstacle detection, or analysis of sound flow, for example when the subject moves or objects move around the subject. Similarly, it may be pertinent to investigate whether special training or substitution devices, frequently described in the literature, not only improve the relevant behavior but also facilitate cross-modal plasticity.

## Materials and Methods

### 

#### Subjects.

The participants were seven healthy sighted volunteers and 12 early-blind subjects who had lost their vision before puberty, most of them in the first few years of life (see Table 2). In each case, the visual deficit was of peripheral origin and led to total blindness except for some light perception in a few subjects (categories 4 and 5, according to the World Health Organization classification [69]). All participants underwent audiometric testing to ensure good hearing, equal in both ears. They gave their written informed consent in accordance with guidelines approved by the Ethics and Research Committees of the Montreal Neurological Institute and the Nazareth and Louis Braille Institute for the Blind.

**Table 2:**
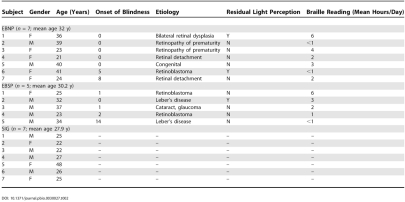
Characteristics of Blind and Sighted Subjects

#### Anechoic chamber experiments.

Subjects were asked to localize sounds binaurally or monaurally while they were seated in the anechoic room. The acoustic apparatus used to test sound localization, previously described in detail [70], consisted of 16 loudspeakers mounted on a graduated semicircular perimeter with a radius of 50 cm (positions: ±5°, ±16°, ±26°, ±37°, ±47°, ±58°, ±68°, and ±78°). The subject was seated in the center of the perimeter, the head placed on a headrest attached to the chair with the speakers positioned at ear level.

The stimuli were broadband noise bursts that lasted 30 ms (10-ms rise and fall times, and a 10-ms plateau). The sound pressure level (SPL) was maintained at 40 dB. A stimulus was delivered through a randomly selected loudspeaker and repeated five times for each position. A buzzer warned the subjects that a sound was about to be presented and that they should maintain a stable head position and fixate straight ahead. Compliance with all instructions was ascertained by an experimenter remaining in the chamber behind the subject. The response consisted of pointing with the dominant hand toward the apparent source of stimulation. Lines graduated in 1° steps were drawn on the perimeter, and the response of the subject was recorded by the experimenter.

For monaural testing, one ear was plugged with a combination of an ear plug (mean attenuation = 37.5 dB SPL) and a hearing protection muff (mean attenuation = 29 dB SPL). In order to compare the overall accuracy in localization between the subjects, the absolute error score was utilized in both the anechoic chamber and scanner experiments. This value is the average of the difference (in absolute value) between the correct position and the response for each trial. To allow combining of data from subjects with left or right ear plugged, the behavioral results were transformed such that the left side was arbitrarily assigned to correspond to the obstructed ear. Thus, in the behavioral data presented, negative angles on the abscissa correspond to the obstructed side, while positive angles correspond to the unobstructed side.

#### Scanner experiments.

In this part of the experiment, subjects were asked to localize sounds binaurally or monaurally while they were lying within the scanner. Monaural testing was carried out using the same ear attenuation procedure as used in the anechoic chamber. All conditions, localization tasks, and their specific control task, were part of a larger study. These conditions were counterbalanced across subjects for the order of scan conditions. Approximately half of the subjects within each group received the ear plug in the left and the other half in the right ear during the monaural part of the scanning session. Auditory stimuli were presented using a circular array of nine speakers, positioned 15° apart from ±60°, and having a radius of 24 cm [31]. The array was placed inside the PET scanner such that the head was in the center of the array, with speakers positioned on the horizontal plane relative to the subject's head, at the level of the ears. In order to ensure stable head position, the head was maintained by a Velcro band, and its position was checked frequently by means of three laser pointers included in the scanner. Background noise in the scanner room was 56 dB SPL.

The stimuli were two broadband noise bursts that lasted 30 ms and were separated by a 0.5-s intrapair interval while the intertrial interval was 2.5 s. Each pair of stimuli was presented from a single speaker at 60 dB SPL, as measured at the center of the array. Each of the nine speakers was utilized 12 times in random order for a total of 108 trials for each condition. The behavioral tasks were started around 15 s before the beginning of data acquisition with the scanner. The response consisted of pointing with a joystick, placed at the subject's side, to the apparent source of stimulation. It was ascertained before the experiments that all subjects were familiar with the use of the joystick and with the task requirements. In a series of preliminary experiments, it was verified that subjects (*n* = 6) wearing binaural earplugs and ear muffs could not localize any of the stimuli from the speakers. The control task consisted of pointing in alternation to the left and right (−90°, +90°, −90°, and so on), after hearing a stimulus pair presented always in the frontal (0°) position. Two control tasks were tested, one binaural and one monaural; in the monaural case the same ear was plugged as was used for the localization task for that individual. Thus, four conditions were tested in all subjects:BSL, binaural control task, MSL, and monaural control task.

PET scans were obtained with a Siemens Exact HR+ tomograph (Forchheim, Germany) operating in three-dimensional acquisition mode. The distribution of CBF was measured during each 60-s scan using the H_2_O^15^ water bolus method [71]. MRI scans (160 1-mm slices) were also obtained for each subject with a 1.5T Philips ACS system (Andover, Massachusetts, United States) to provide anatomical detail. CBF images were reconstructed using a 14-mm Hanning filter, normalized for differences in global CBF, and co-registered with the individual MRI data [72]. Each matched MRI/PET dataset was then linearly resampled into a standardized stereotaxic coordinate system based on the MNI305 target (a sample of 305 normal subjects) via an automated feature-matching algorithm [73], resulting in a normalized brain space similar to the Talairach and Tournoux atlas (for additional information, see: http://www.mrc-cbu.cam.ac.uk/Imaging/). Statistical analysis was performed applying the method described by Worsley et al. [74]; covariation analysis followed the procedure outlined by Paus et al. [33]. A *t* value of 3.5 was used for significant changes in CBF during exploratory searches. However, a *t* value of 3.0 was used for a priori regions of interest, such as occipital areas.

## Supporting Information

Figure S1 Parietal Activation Foci in Binaural Sound Localization Task Sagittal and coronal images contrasting BSL to the control task. All three groups showed increased CBF in the right inferior parietal lobe (as shown by the red arrows), consistent with other neuroimaging studies of auditory localization. X and Y coordinates refer to standardized stereotaxic space.
(2.2 MB TIF).

Table S1 Stereotaxic Coordinates and *t* Values of Activation and Deactivation Foci (35 KB DOC).

## Abbreviations

BA: Brodmann area
BSL: binaural sound localization
CBF: cerebral blood flow
EBNP: early blind with normal performance
EBSP: early blind with superior performance
MSL: monaural sound localization
PET: positron emission tomography
SIG: sighted control group
SPL: sound pressure level
