# Supplementary figures and images for: A Functional Neuroimaging Study of Sound Localization: Visual Cortex Activity Predicts Performance in Early-Blind Individuals
Source: PLoS Biol. 2005 Jan 25;3(2):e27. doi: 10.1371/journal.pbio.0030027 (PMC544927; doi:10.1371/journal.pbio.0030027)

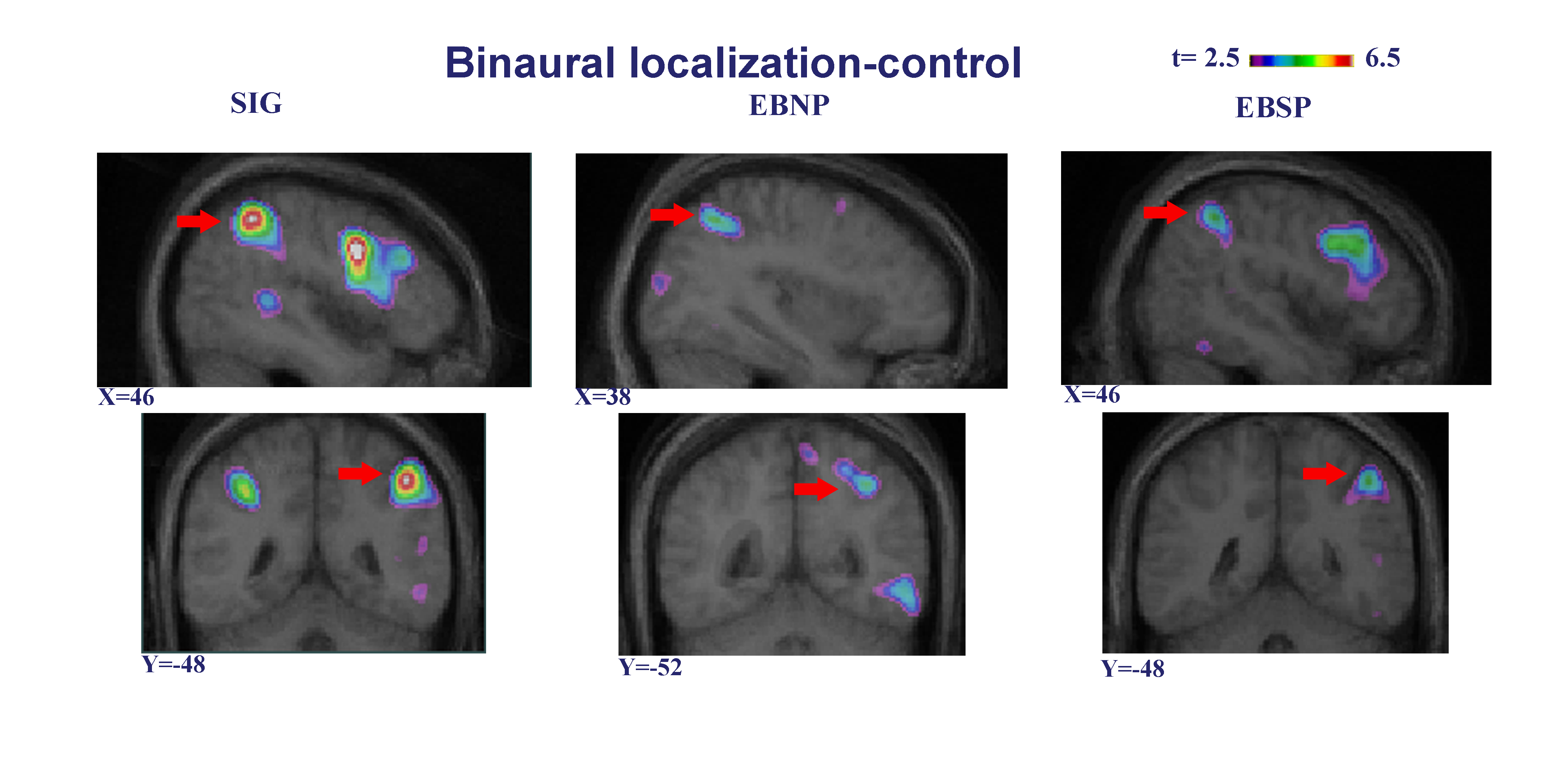

Supplement: Figure S1 — Sagittal and coronal images contrasting BSL to the control task. All three groups showed increased CBF in the right inferior parietal lobe (as shown by the red arrows), consistent with other neuroimaging studies of auditory localization. X and Y coordinates refer to standardized stereotaxic space. (2.2 MB TIF). [file pbio.0030027.sg001.tif]
