# Supplementary material for: A Functional Neuroimaging Study of Sound Localization: Visual Cortex Activity Predicts Performance in Early-Blind Individuals
Source: PLoS Biol. 2005 Jan 25;3(2):e27. doi: 10.1371/journal.pbio.0030027 (PMC544927; doi:10.1371/journal.pbio.0030027)
Supplement: Table S1 — (35 KB DOC). [file pbio.0030027.st001.doc]

## Table 1. Stereotaxic Coordinates and t-values of Activation and Deactivation Foci

Binaural sound localization (BSL)

minus baseline:

Area categories Groups Areas x y z t

Occipital SIG R Cuneus 7 -88 29 -4.88

L Cuneus (V1) -13 -99 3 -4.36

R Lingual gyrus 3 -78 2 -4.06

L Lingual gyrus -21 -78 -9 -3.95

EBNP

EBSP R Lingual gyrus 16 -83 -14 3.88

Parietal SIG R Inferior parietal lobule 46 -49 48 6.03

L Precuneus -8 -71 56 5.16

L Inferior parietal lobule -36 -50 44 4.89

R Superior parietal lobule 20 -73 53 4.31

L Postcentral gyrus -35 -31 63 -4.12

EBNP R Superior parietal lobule 27 -61 53 4.87

R Superior parietal lobule 15 -66 53 4.76

L Superior parietal lobule -16 -61 56 4.04

EBSP R Superior parietal lobule 17 -74 39 4.29

L Precuneus 1 -76 44 4.24

R Inferior parietal lobule 46 -49 48 4.10

R Inferior parietal lobule 52 -44 41 3.75

L Postcentral gyrus -38 -38 60 -4.83

Temporal SIG L Middle temporal gyrus -59 -40 -9 3.68

R Superior temporal gyrus 54 -42 15 3.65

R Middle temporal gyrus 44 -40 6 3.59

L Middle temporal gyrus -43 -1 -18 -3.67

EBNP R Inferior temporal gyrus 51 -61 -6 4.57

L Parahippocampal gyrus -17 -26 -15 -3.78

EBSP R Inferior temporal gyrus 51 -55 -19 3.85

L Parahippocampal gyrus -31 -25 -18 -3.82

Frontal SIG R Precentral gyrus 44 5 33 6.44

R Inferior frontal gyrus 40 18 12 4.29

R Middle frontal gyrus 27 44 -12 4.28

R Middle frontal gyrus 43 29 29 4.04

R Middlefrontal gyrus 27 -6 57 3.98

L Inferior frontal gyrus -43 15 6 3.85

R Medial frontal gyrus 4 8 51 3.61

L Superior frontal gyrus -15 49 14 -3.86

L Precentral gyrus -17 -30 65 -3.63

EBNP L Medial frontal gyrus -1 10 53 3.77

R Superior frontal gyrus 13 55 17 -4.00

L Medial frontal gyrus -5 34 -12 -3.96

L Superior frontal gyrus -9 46 44 -3.52

EBSP R Middle frontal gyrus 43 13 35 4.69

R Middle frontal gyrus 44 24 33 4.49

R Middle frontal gyrus 27 58 -9 3.88

R Medial frontal gyrus 7 18 47 3.76

R Precentral gyrus 35 -7 47 3.52

R Medial frontal gyrus 4 -14 60 -5.64

R Precentral gyrus -38 -18 62 -5.38

L Precentral gyrus -11 44 2 -4.66

Others SIG R Putamen 23 5 8 4.05

R Anterior intern capsule 7 -16 -21 3.82

L Cingulate region -1 -59 17 -5.00 L Cingulate region -8 46 3 -3.70

L Cingulate region -11 -47 30 -3.70

EBNP L Cingulate region -4 46 -8 -4.20

R Cingulate region 4 -52 29 -4.08

EBSP L Cerebellum -7 -56 -15 -3.97

Monaural sound localization (MSL)

minus baseline:

Area categories Groups Areas x y z t

Occipital SIG

EBNP

EBSP

R Cuneus (V1) 13 -81 15 4.14

R Lingual gyrus 15 -73 -6 3.39

Parietal SIG R Superior parietal lobule 8 -64 53 4.86

L Precuneus -3 -69 51 4.19

R Inferior parietal lobule 48 -50 45 3.64

L Inferior parietal lobule -51 -61 33 -4.05

EBNP R Superior parietal lobule 19 -66 53 4.77

R Inferior parietal lobule 43 -52 51 3.95

L Superior parietal lobule -12 -71 53 3.61

R Postcentral gyrus 25 -30 57 -4.54

R Postcentral gyrus 42 -16 48 -4.41

L Precuneus -4 -49 39 -4.15

L Precuneus -11 -61 18 -3.93

EBSP R Superior parietal lobule 21 -69 44 4.25

L Precuneus -3 -76 51 4.19

R Superior parietal lobule 31 -68 51 3.78

L Postcentral gyrus -43 -19 53 -3.78

R Postcentral gyrus 24 -37 59 -3.55

Temporal SIG

EBNP L Parahippocampal gyrus -20 -31 -20 -4.12

R Parahippocampal gyrus 21 -13 -20 -3.66

R Middle temporal gyrus 59 3 -20 -3.57

EBSP

Frontal SIG R Middle frontal gyrus 28 -1 56 4.70

R Medial frontal gyrus 4 8 57 4.56

R Middle frontal gyrus 40 8 36 4.53

R Middle frontal gyrus 27 53 -11 4.13

R Inferior frontal gyrus 52 10 21 3.69

L Cingulate region -1 37 -5 -4.57

L Cingulate region -1 27 14 -4.16

L Medial frontal gyrus 0 55 20 -4.15

L Medial frontal gyrus -3 58 8 -4.06

L Uncus -29 -7 -18 -3.91

L Precentral gyrus -34 -19 63 -3.91

L Precentral gyrus -17 -25 65 -3.79

EBNP R Medial frontal gyrus 7 24 41 4.82

R Middle frontal gyrus 43 46 8 4.57

R Middle frontal gyrus 43 44 18 4.53

R Middle frontal gyrus 25 3 54 4.39

L Middle frontal gyrus -34 49 21 3.77

L Inferior frontal gyrus -46 17 12 3.54

R Insula 31 18 2 3.52

R Precentral gyrus 5 -21 54 -4.95

R Medial frontal gyrus 3 49 33 -4.28

L Medial frontal gyrus -4 55 5 -3.91

L Precentral gyrus -25 -26 51 -3.82

EBSP R Superior frontal gyrus 4 22 50 3.88

L Medial frontal gyrus -7 -11 57 -3.99

L Medial frontal gyrus -3 61 23 -3.61

L Superior frontal gyrus -8 44 39 -3.55

Other regions SIG L Cingulate region -1 -56 23 -5.91

EBNP Brain stem -5 -23 -18 3.60

EBSP R Cerebellum 7 -80 -20 5.03

R Cerebellum 3 -71 -21 4.80

R Posterior limb of internal capsule 15 12 0 3.68

L Anterior internal capsule -16 -11 -12 -3.90

L Cingulate region -1 44 -9 -3.69

Covariation with behavioral measure (absolute error) , early blinds only:

a)BSL minus baseline

Area Categories Areas x y z t

Occipital L Cuneus (V1) -13 -79 9 -3.35

Parietal

oral

Temp

Frontal L Precentral gyrus -9 -26 62 3.68

R Medial frontal gyrus 42 24 29 -3.53

b)MSL minus baseline

Area categories Areas x y z t

Occipital R Lingual gyrus 12 -67 -6 -5.13 R Superior occipital gyrus 46 -64 17 -4.01

R Cuneus (V1) 3 -83 12 -3.12

R Cuneus (V1) 17 -93 2 -3.09

R Middle occipital gyrus 42 -76 32 -3.01

Parietal L Superior parietal lobule -24 -41 44 3.58

Temporal L Inferior temporal gyrus -59 -57 -16 3.76

R Middle temporal gyrus 44 -53 9 -3.57

Frontal L Inferior frontal gyrus -52 7 29 3.81

R Medial frontal gyrus 13 3 53 3.77

Coordinates refer to standardized stereotaxic space [75]
